# Advanced Neuroimaging and Clinical Findings in a Case of Linear Scleroderma En Coup De Sabre Presenting With Seizures

**DOI:** 10.7759/cureus.74687

**Published:** 2024-11-28

**Authors:** Yukiyoshi Kimura, Dafne Soto-Trujillo, Karen Fernandez-Leon

**Affiliations:** 1 Radiology, Grupo CT Scanner, Mexico City, MEX

**Keywords:** en coup de sabre, localized scleroderma, mri- magnetic resonance imaging, neuro-imaging, neurology and epilepsy

## Abstract

Scleroderma is a rare connective tissue disease categorized as systemic or localized. Linear subtype of localized scleroderma usually manifests as a cutaneous linear scar-like lesion most commonly on the scalp. It may present with neurologic, ophthalmologic, and rheumatologic symptoms. We present a case of a 24-year-old female patient with a band-like scalp brown sclerotic lesion in the left upper frontal region presenting with headaches and a first-time seizure who underwent 3.0-T magnetic resonance and described the structural and advanced neuroimaging findings, including perfusion and spectroscopy, that may help narrow differential diagnosis of linear scleroderma.

## Introduction

Scleroderma is a rare connective tissue disease categorized as systemic or localized [1]. Localized scleroderma is classified into five subtypes: linear, circumscribed, generalized, pansclerotic, and mixed [2]. The linear subtype is usually diagnosed in pediatric age, up to 67% of cases, and occurs more frequently in white females. It usually manifests as a cutaneous linear scar-like lesion with alopecia in the affected area [3]. This subtype commonly affects the scalp and can extend to the nose, jaw, cheeks, and neck, but it may also involve the limbs [4]. “En coup de sabre” linear scleroderma (ECDS) refers to cases involving the forehead: frontoparietal region. ECDS is a rare subtype with an incidence of 0.4 to 2.7 per 100,000 [5]. Initially thought to be limited to the skin, ECDS may also present with neurologic, ophthalmologic, and rheumatologic symptoms. Epilepsy is the most common neurologic symptom; however, patients may also experience movement disorders and behavioral changes [6]. Neurological symptoms are a strong predictor of positive characteristic ipsilateral magnetic resonance imaging (MRI) findings. Positive radiological findings such as ipsilateral brain atrophy, calcifications, and T2/FLAIR hyperintense white matter changes have been reported [7]. Positive imaging findings may also be seen in asymptomatic patients [8].

In this case report we describe the case of a young adult woman with ECDS with clinical and radiological neurologic manifestations.

## Case presentation

**Figure 1:**
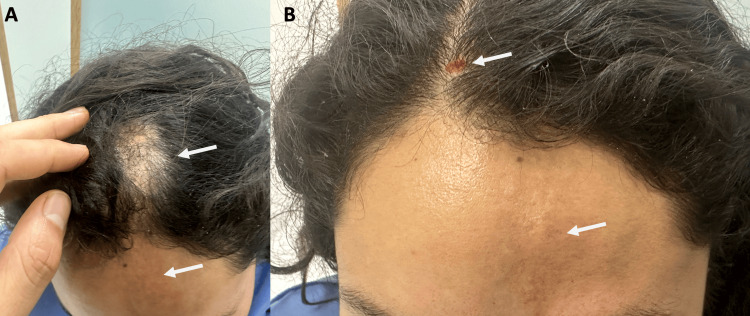
Patient’s photographs show cicatricial alopecia in the frontal scalp and paramedian forehead (A), associated with focal scalp hypotrophy with skin color changes and sclerosis at the left frontal region with a linear configuration (B)

A young 24-year-old Latin-American female was referred to the radiology department for a brain MRI with and without contrast by the neurology department for presenting a first unprovoked complex partial seizure. The patient had a non-significant past medical, family, or surgical history. She only referred headaches starting at age 13 with increased intensity over time. On physical examination, the patient had a band-like sclerotic lesion in the left upper frontal region, with a red plaque and focal volume loss, associated with focal frontal alopecia in this region, which she noted since she was 11 (Figure 1). 

**Figure 2:**
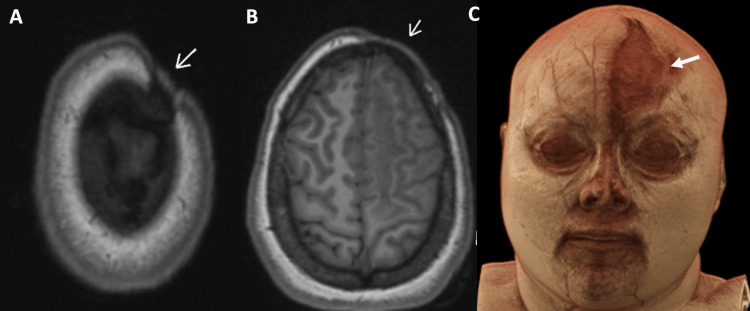
T1 MPRAGE images (A and B) and volumetric rendered reconstruction (C) show focal scalp hypotrophy with sclerosis in the left fronto-parietal region in a linear configuration (arrow) MPRAGE: magnetization prepared rapid gradient echo

**Figure 3:**
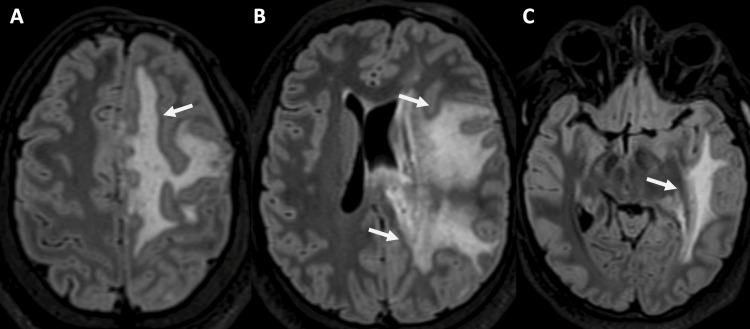
Volumetric T2 FLAIR axial images in the dorsoventral direction (A-C) show subcortical white matter signal abnormalities involving the left fronto-parieto-temporal lobe and the splenium of the corpus callosum (arrows)

**Figure 4:**
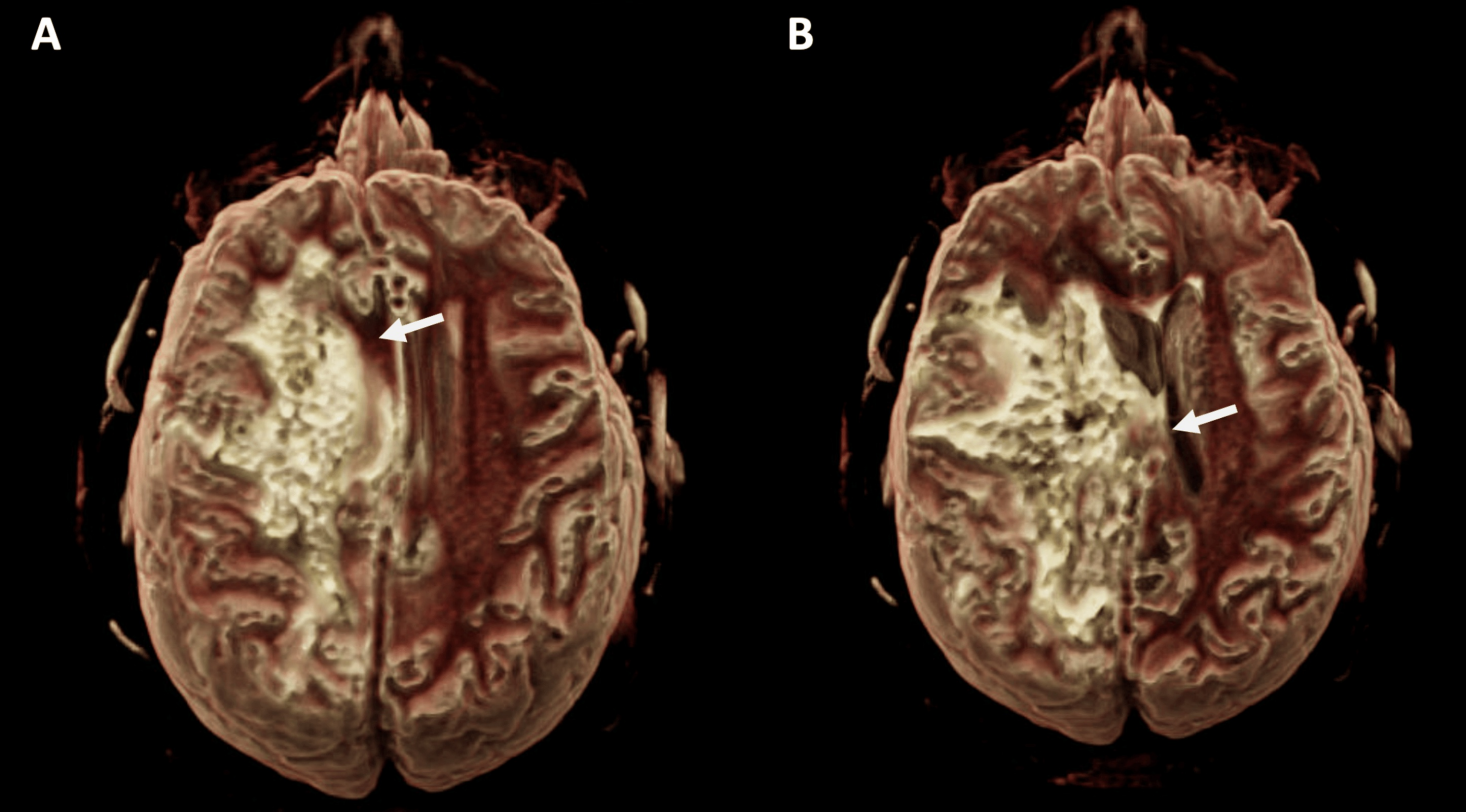
Cinematic-rendered volumetric T2 FLAIR images (A and B) show subcortical white matter signal abnormalities in the left cerebral hemisphere (arrows). The patient is seen from the back

**Figure 5:**
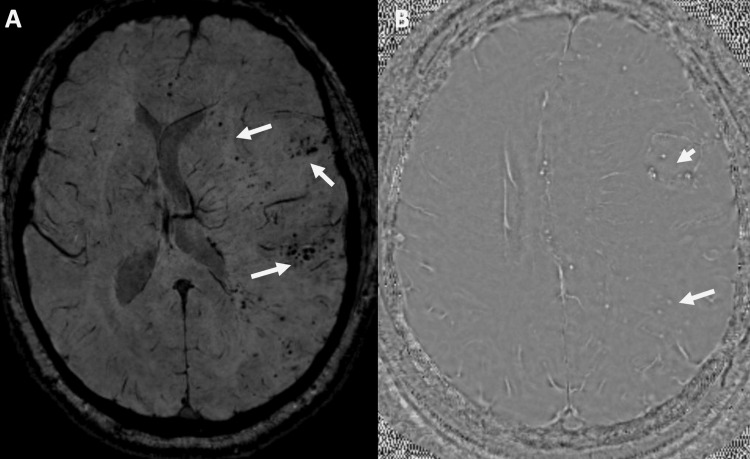
Susceptibility-weighted image (A) and phase mask (B) show several small focal blooming artifacts that are hyperintense on the phase mask of a left-hand system, suggesting microhemorrhages (hemosiderin)

A brain MRI with and without contrast was performed on a 3-T MR Scanner (Lumina, Siemens Healthcare, Erlangen, Germany) using a dedicated 20-channel coil. Imaging demonstrated, on volumetric T1 images, focal scalp atrophy with minimal frontal bone flattening (Figure 2), subcortical white matter changes in the ipsilateral frontal-parieto-temporal lobe (Figures 3, 4), and blooming artifacts on susceptibility-weighted images with hyperintensities on the phase mask (left-hand MR system), suggesting microhemorrhages (Figure 5).

**Figure 6:**
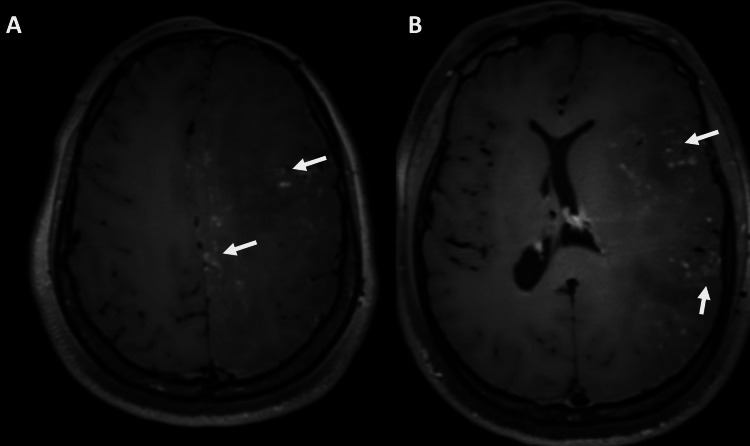
Sub-volumetric (0.7 mm) T1 SPACE images show nodular, curvilinear enhancement at the convexity and at the level of the basal ganglia

After contrast administration, nodular, curvilinear enhancement at the convexity and at the level of the basal ganglia was observed (Figure 6).

**Figure 7:**
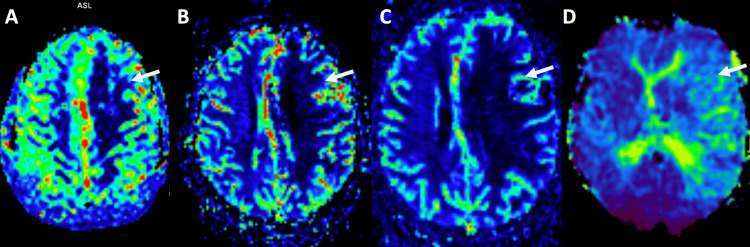
Relative cerebral blood flow map (A), acquired using an arterial spin labeling perfusion technique, and relative cerebral blood volume maps (B and C), acquired during contrast administration with a dynamic susceptibility contrast technique, shows decreased perfusion in the left fronto-parietal lobes with a slight increase in mean transit time (D)

On advanced MRI sequences, the relative cerebral blood flow was diffusely decreased on arterial spin labeling perfusion. Relative cerebral blood volume and blood flow were also decreased on dynamic susceptibility contrast perfusion, with a slightly increased mean transit time (Figure 7).

**Figure 8:**
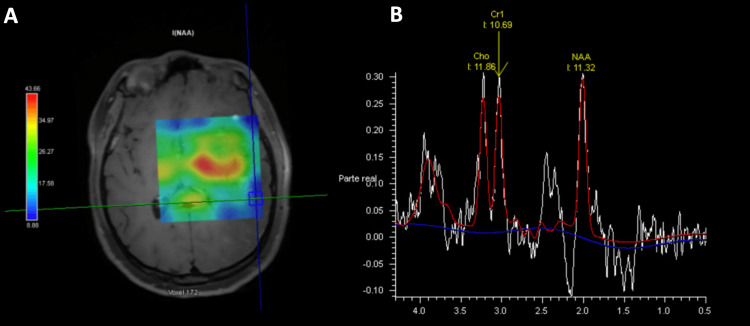
Short echo time color NAA (A) shows decreased NAA in the subcortical white matter signal intensity abnormality. The MR spectroscopy curve (B) shows a slight increase in choline concentration (11.86) and a decrease in NAA concentration (11.32) NAA: N-Acetyl-Aspartate color map

On MR spectroscopy acquired with a short echo time (40 ms), there was a slight increase in the choline concentration and a decrease in N-Acetyl-Aspartate color map (NAA) concentration (Figure 8).

## Discussion

Localized scleroderma is a rare connective tissue disease of unknown etiology. It is a distinct and separate disease entity from systemic scleroderma. Clinically it is differentiated from systemic sclerosis by the absence of Raynaud’s phenomenon, sclerodactyly, and erythema. Linear scleroderma ECDS is a subtype of localized scleroderma. 

The diagnosis of linear scleroderma ECDS is clinical with no definite imaging, serologic, or pathologic pathognomonic features. It is more frequent in women at menarche (12-15 years of age) and in the fifth decade of life.

Linear scleroderma has been associated with several neurologic abnormalities in around 20% of the patients, usually preceded by the development of cutaneous disease in months to years [9,10]. Neurologic symptoms can present after years of cutaneous manifestations; however, they can appear first in 16% of cases [9]. Physio-pathologic mechanisms regarding the central nervous system include infiltration of perivascular lymphocytes in angio-inflammatory lesions causing abnormal and dilated blood vessels, astrocyte hyperplasia leading to brain parenchyma, meninges, and vascular scarring and local ischemia by destruction of endothelial cells, fibroblast activation and collagen contraction [11-13].

Neurological symptoms with linear scleroderma are variable. The most common neurological manifestations are epilepsy (seen in 73% of patients, 33% of them refractory to treatment), focal neurological deficits, headaches (35% of patients), and movement disorders (11% at presentation and 35% of patients overall). Less prevalent neurological manifestations include trigeminal neuralgia, hemiplegic migraine, behavioral changes, and neuropsychiatric symptoms [6].

CT and MRI have shown central nervous system findings, most of them ipsilateral to the cutaneous lesion. A study by Nadeau, et al. found that 78% of 55 symptomatic patients with linear scleroderma had positive imaging findings only on the side of the skin lesion [14].

Most symptomatic patients have abnormalities in imaging studies; however, patients with positive imaging findings may be asymptomatic [15]. The most prevalent imaging findings are white matter abnormalities, which most commonly affect a single lobe but can also be diffuse and confluent, with multilobar involvement, as seen in our patient. Other imaging findings include subtle brain volume loss with sulcal widening, ex vacuo dilation of the ventricles, cortical thickening, nodular and curvilinear enhancement, and ipsilateral intraparenchymal calcifications, most commonly in the thalami, dentate nuclei, and basal ganglia [9].

Focal scalp and less often diploe thinning at the fronto-parietal region can also be seen on imaging studies. Vascular involvement has been described on MR angiograms and cerebral angiographies, which suggest a vasculitis process. Some pathologic specimens point to lymphocytic vasculitis as part of the underlying etiology [16].

Our patient presents with diffuse microhemorrhages limited to the white matter, with signal intensity changes represented by punctate blooming artifacts on susceptibility-weighted imaging. To our knowledge, intracranial hemorrhage is a rare imaging finding on ECDS. Ipsilateral cavernomas have been reported previously in ECDS by Fain, et al. [17]. Microhemorrhages have also been reported in a case of progressive facial hemiatrophy (Parry-Romberg syndrome), which is a related variant within the scleroderma spectrum of disease [18,19]. 

Limited information regarding advanced MRI sequences has been described. MRI dynamic susceptibility contrasts perfusion on previous case reports showed decreased relative cerebral blood flow and volume and increased mean transit time at the white matter signal abnormality, such as in our case [1]. Arterial spin labeling perfusion also showed decreased relative cerebral blood flow. These findings correlate with the nonspecific hypometabolism seen in 18F-FDG PET studies. At MRI spectroscopy our case shows similar findings to the ones described by Nguyen, et al. with a discrete increase in choline (3.5 ppm) concentration and a decrease in N-Acetyl-Aspartate (2.0 ppm) concentration [1]. These advanced imaging findings are nonspecific but useful in narrowing the differential diagnosis, differentiating ECDS from high-grade gliomas and other malignant processes.

## Conclusions

Linear scleroderma ECDS is a rare connective tissue disease. There are no pathognomonic imaging or serological findings. However, certain MRI findings such as focal scalp atrophy, ipsilateral white matter hyperintensities, low relative cerebral blood flow/volume associated with increased mean transit time, and nodular/curvilinear enhancement in conjunction with clinical examination may assist in differential diagnosis.

Advanced imaging sequences, such as MRI perfusion and spectroscopy, may be useful in differentiating central nervous system localized scleroderma infiltrative involvement from high-grade gliomas. 
